# Use of the Trauma and Injury Severity Score (TRISS) as a Predictor of Patient Outcome in Cases of Trauma Presenting in the Trauma and Emergency Department of a Tertiary Care Institute

**DOI:** 10.7759/cureus.40410

**Published:** 2023-06-14

**Authors:** Shubham K Indurkar, Pankaj S Ghormade, Swapnil Akhade, Bedanta Sarma

**Affiliations:** 1 Forensic Medicine and Toxicology, All India Institute of Medical Sciences, Raipur, IND; 2 Forensic Medicine, All India Institute of Medical Sciences, Raipur, IND; 3 Forensic Medicine, All India Institute of Medical Sciences, Mangalagiri, IND

**Keywords:** mortality, road traffic accidents, triss, rts, iss, ais, trauma

## Abstract

Background: In this study, we used the anatomic scoring system Abbreviated Injury Scale (AIS) to calculate the Injury Severity Score (ISS) and the physiological scoring system for the Revised Trauma Score (RTS) on the arrival of patients. Both scores were used to calculate the Trauma and Injury Severity Score (TRISS) for predicting the patient outcome in a case of trauma.

Methods: This prospective, cross-sectional, observational study was carried out at the trauma centre of a tertiary care institute and included patients of either sex, age ≥18 years, and ISS ≥15. A total of 2084 cases of trauma over a period of 18 months were assessed, and 96 cases of blunt trauma meeting the inclusion criteria were studied.

Results: Patients injured in road traffic accidents constituted the maximum caseload. Out of a sample size of 96 patients with ISS ≥15, 77 died during the treatment and 19 survived.

The ISS ranged from 15 to 66, with a mean ± SD score of 27.48 ± 8.79. Non-survivors had a statistically higher significant ISS than survivors (p<0.001). The RTS ranged from <1 to 7.84, with a mean ± SD score of 4.52 ± 2.08. Non-survivors had low RTS (RTS <5, n=52) compared to survivors, and the difference was statistically significant (p<0.001).

The mean ± SD TRISS (Ps) score was 0.69 ± 2.288. In the non-survivor (NS) group, 15 patients had TRISS (Ps) between 0.26-0.50, followed by 0.51-0.75 (n=18), 0.76-0.90 (n=12), and 0.90-0.95 (n=11). While 16 survivors had TRISS (Ps) between 0.96 and 1, a statistically significant association was found between TRISS and patient outcome (p-value <0.001).

On the receiver operating characteristic (ROC) curve analysis, the sensitivity of TRISS (94.7%) and RTS was found to be comparable (94.7%), whereas ISS was less sensitive (36.8%) in predicting the patient outcome. RTS (79.2%) and TRISS (76.6%) scores were more specific than ISS (5.2%) for outcome analysis.

Conclusion: The TRISS score is useful in the management of trauma patients as it can satisfactorily predict mortality in a case of trauma. The trauma scores are of immense help in determining the nature of injury in medicolegal cases.

## Introduction

Trauma has been known as a worldwide cause of death and disability for decades now [1]. This burdens society with a lot of direct and indirect socioeconomic costs [2].

Various modalities of trauma, like road and railway incidents, falls, violence and assaults, self-inflicted injuries, burns, etc., kill around five million people all around the globe annually, accounting for almost 9% of worldwide mortality [3]. Globally, a significant proportion of deaths following trauma are due to road traffic accidents (RTA), causing an enormous burden in the healthcare sector [4].

According to the National Crime Records Bureau (NCRB) report published in 2019, in India, there were a total of 549,370 accidents (including air crashes, ship accidents, structure collapse, drowning, electrocution, explosions, falls, workplace injury, fire, firearm, mine accidents, and traffic accidents) in 2019, causing 445,420 injured patients and 263,031 deaths. Out of these, there were 467,171 reports of traffic accidents (both road and railway), leaving behind 442,996 injured cases and 181,113 deaths [5].

A quantitative method for measuring trauma severity has many potential applications: patient triage, common terminology about injury severity, prognosis assessment, trauma care audit, and epidemiology [6]. Tools like trauma scores precisely quantify the severity of trauma and outcome probability, which are critical in understanding the gap between problems and the definitive management of trauma patients [7]. Trauma scores are beneficial in framing opinions regarding the nature of the injuries in medico-legal cases [7,8]. Forensic medicine experts are routinely called upon to examine injuries in hospitalised trauma patients. Hence,it is necessary to assess the outcome of the injuries before labelling the nature of the injuries as grievous or not under Section 320 of the Indian Penal Code (IPC) [9].

Trauma scores can be categorised as anatomical (Abbreviated Injury Scale, Injury Severity Score), physiological (Revised Trauma Score), or combined (Trauma and Injury Severity Score). Physiological scores can be measured at the initial clinical assessment of the patient, whereas anatomical scoring of the injuries can be easily done later on after stabilising the patient. Hence, the stratification of trauma patients can be easily done [10]. On the other hand, combined scores that include both anatomical and physiological criteria are more useful for patient prognosis. One such combined score is the Trauma and Injury Severity Score (TRISS), which was designed by the Major Trauma Outcome Study (MTOS) in the United States to predict the outcome in polytrauma patients and includes the Injury Severity Score (ISS) and Revised Trauma Score (RTS) [11].

In the Indian context, only a few studies have demonstrated the use of different trauma scores to predict outcomes in patients with trauma, especially TRISS [10,12,13]. Hence, this study was undertaken with the aim of filling in this gap and trying to examine the utility of TRISS scores to estimate patient outcomes in cases of trauma, followed by setting up a triage and trauma management protocol.

## Materials and methods

This prospective cross-sectional observational study was carried out over a period of 18 months, from May 2020 to November 2021, at the Trauma and Emergency Department of the All India Institute of Medical Sciences (AIIMS), Raipur, India.

We had included patients of either sex, aged ≥18 years, with an Injury Severity Score (ISS) ≥15 requiring hospitalisation. Cases of trauma brought dead on arrival to the hospital, those who took discharge against medical advice (DAMA), absconded, were referred from other hospitals after a major surgical initial intervention, and the patient or relatives not consenting or willing to participate in the study were excluded from the study. We used convenient sampling and included all consenting trauma patients, including criteria.

Collection of data

Demographic details and the condition of the patient on arrival were noted in a validated case record form (CRF), followed by the calculation of trauma scores. Injuries were documented in the standard prescribed Abbreviated Injury Scale (AIS) scoring and used to calculate the ISS. The Revised Trauma Score (RTS) and ISS were calculated after the initial assessment of the patient, followed by the calculation of the TRISS score. Each patient was then followed up during the hospital stay until the final outcome (death or discharge). All the data were compiled for outcome prediction in TRISS, and a comparative analysis of trauma scores was done to predict outcomes in patients (Table 1).

**Table 1:** Calculation of trauma scores

Formulae for Trauma Scores
1. Injury Severity Score (ISS): Three of the most severely injured different body regions as per the Abbreviated Injury Scale (AIS) were selected for the calculation of ISS: ISS ISS= X^2^+ Y^2^+Z^2 [14]^
2. Revised Trauma Score (RTS): RTS=0.9368(GCSc)+0.7326(SBPc)+0.2908(RRc) ^[15]^
3. The Trauma and Injury Severity Score (TRISS) was calculated as: b=b0+b1(RTS)+b2(ISS)+b3(Age Index) ^[16]^ Probability of survival for TRISS (Ps)- Ps=1+e-b ^[16]^ Abbreviations- bo, b1, b2, b3 were the respective coefficients used for blunt and penetrating trauma; Ps: probability of survival; age index: 1 if age 55 and zero (0) if age<55; X, Y, Z were the scores from the three most severely injured different body parts as per AIS.

Statistical analysis

Data were entered in Microsoft Excel 13, and all statistical analysis was done in International Business Machines (IBM) Statistical Package for the Social Sciences (SPSS) version 20. Parametric data were presented as mean ± SD and compared by an independent t-test (two groups). Non-parametric data were shown as median and interquartile range and compared by two samples using Mann-Whitney U (two groups). Bivariate correlation (Spearman's correlation coefficient) finds associations between continuous variables. Logistic regression was applied to associate continuous variables with categorical data and positive predictive value, negative predictive value, and accuracy. The receiver operating characteristic (ROC) curve was plotted for trauma scores. ROC statistics were used to calculate sensitivity, specificity, and cut-off values. A p-value of less than 0.05 was taken as statistically significant.

Institutional ethics committee (IEC) approval

The study was approved by the IEC of AIIMS Raipur vide no. 036/IEC-AIIMSRPR/2020 dated May 11, 2020.

## Results

We assessed a total of 2084 cases of mechanical injuries that included 1455 (69.82%) cases of road traffic accidents (RTA), 251 (12.04%) cases of assaults (47 cases of stab and sharp weapon injuries, one case of a firearm, and 208 cases of other blunt injuries), 35 (1.68%) cases of burns or electrocution, and 343 (16.46%) cases of other miscellaneous injuries.

Out of a total of 2084 patients with mechanical trauma, only 96 qualified to be included in the study. All the included cases had blunt trauma with ISS≥15. All the cases of penetrating trauma had ISS<15; hence, they were excluded from the study.

Sex-wise distribution

**Figure 1:**
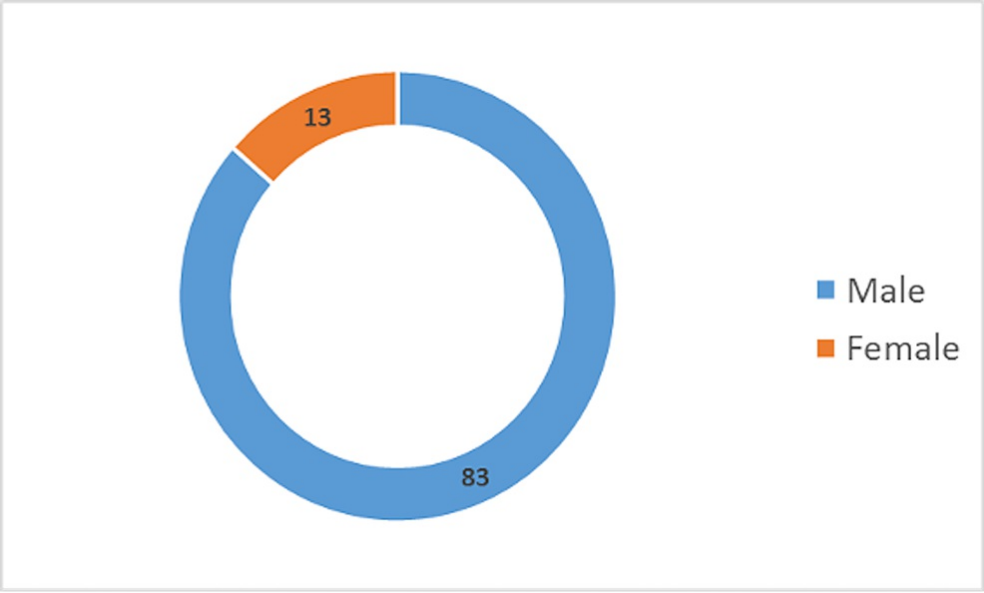
Sex-wise case distribution

As shown in Figure 1, the male-to-female ratio in the study was 6.6:1, with 86.5% being men and 13.5% being women. 

Age-wise distribution

**Figure 2:**
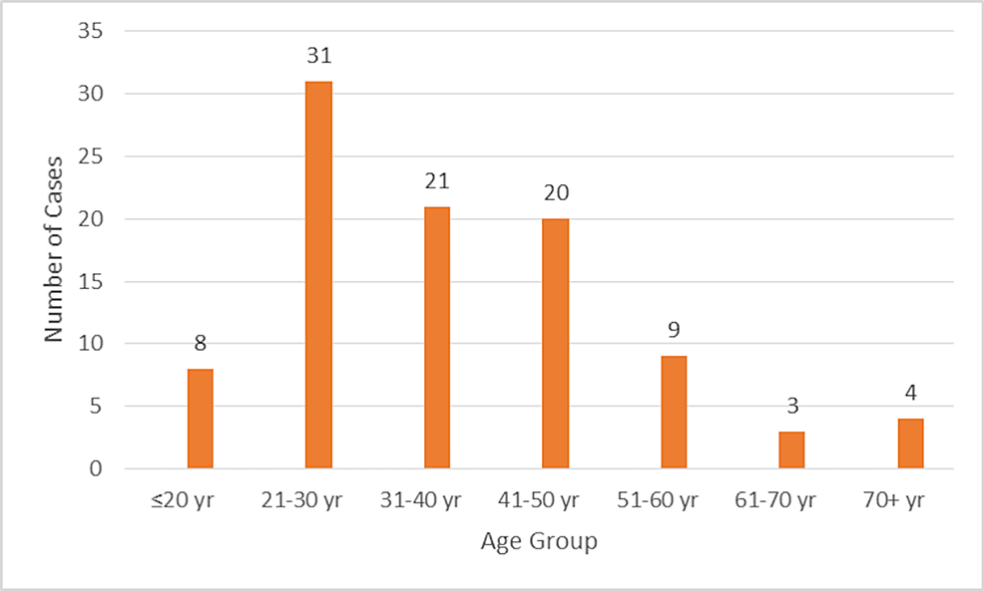
Age-wise distribution of cases

As shown in the bar diagram (Figure 2), the age-wise distribution of cases included in the study ranged from 18 years to 79 years. The mean age ± SD of the study group was 37.46 14.7 years, with the maximum number of patients falling in the age group of 21-30 years (32.3%), followed by 31-40 years (21.9%). In all the age groups, male patients outnumbered female patients.

Outcome of cases

**Figure 3:**
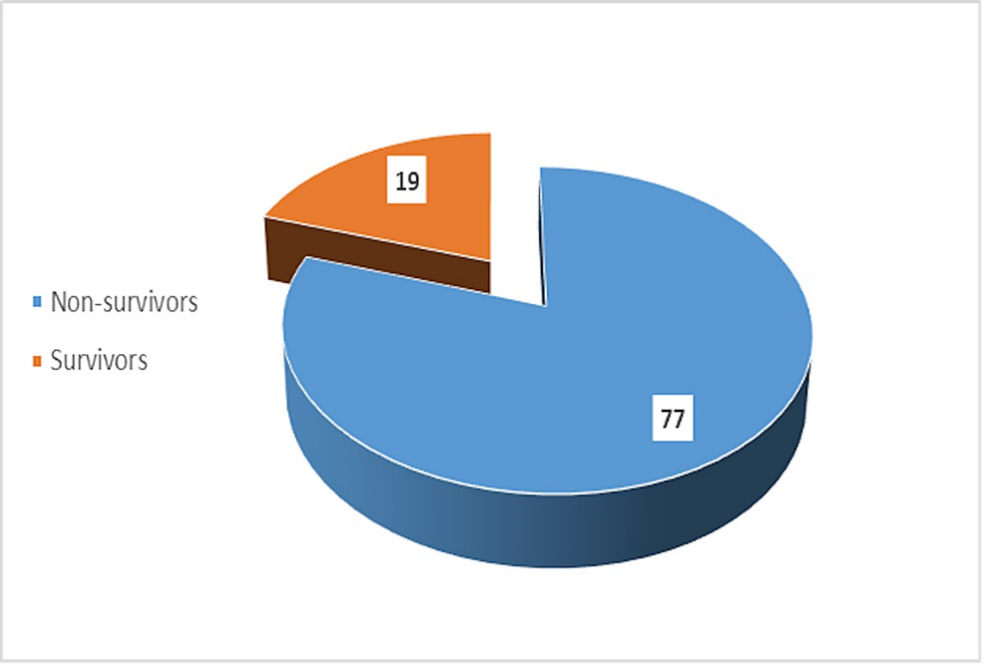
Outcome of cases

Figure 3 depicts a pie diagram showing the outcome of the study of patients. Overall, 19.80% (n=19) of patients survived mechanical trauma, whereas 80.20% (n=77) succumbed to the injuries.

Trauma scores

The trauma scores under study were the Injury Severity Score (ISS), the Revised Trauma Score (RTS), and the Trauma and Injury Severity Score (TRISS). The means and median values of trauma scores are shown in Table 2.

**Table 2:** Means and medians of the trauma scoring system ISS: Injury Severity Score; RTS: Revised Trauma Score; TRISS: Trauma and Injury Severity Score

Scoring systems	Mean ± SD	Median
ISS	27.48 ± 8.79	25 (4)
RTS	4.52 ± 2.08	4.25 (7.8)
RTS %	63.93 ± 31.01	66.45 (53.9)
TRISS	0.69 ± 2.288	0.772 (3.08)
TRISS %	60.71 ± 32.80	68.4 (58.07)

Injury severity score (ISS)

ISS was grouped as shown in Table 3. The ISS ranged from 15 to 66 in our study, with a mean ± SD score of 27.48 ± 8.79 (Table 2). The majority of non-survivors (NS) (n = 63) had ISS between 25 and 40, followed by ISS 41-49 (n=8), whereas a higher number of survivors (S) (n=12) had ISS 16-24, followed by ISS 25-40 (n=6). Non-survivors had a higher ISS than survivors, and the difference was statistically significant (p<0.001).

**Table 3:** ISS vs. patient outcome NS: non-survivors; S: survivors

ISS group	Patient outcome		Total	Pearson's chi-square test	p-value
	NS	S			
16-24	4	12	16	39.398	<0.001
25-40	63	6	69		
41-49	8	0	8		
50-74	2	1	3		
Total	77	19	96		

Revised trauma score (RTS)

RTS scoring was also grouped as shown in Table 4. The RTS ranged from <1 to 7.84, with a mean ± SD score of 4.52 ± 2.08 (Table 2). Patients who died during the treatment had low RTS (RTS <5, n=52) compared to survivors. In contrast, all the survivors (n=19) had RTS>5. A statistically significant association was noted between RTS and patient outcome (p<0.001).

**Table 4:** RTS vs. patient outcome NS: non-survivors; S: survivors

RTS group	Patient outcome		Total	Pearson's chi-square test	p-value
	NS	S			
0 - <1	5	0	5	74.33	<0.001
1 - <2	5	0	5		
2 - <3	13	0	13		
3 - <4	10	0	10		
4 - <5	19	0	19		
5 - <6	23	2	25		
6 - <7	2	8	10		
7 -7.84	0	9	9		
Total	77	19	96		

Trauma and injury severity score (TRISS)

TRISS (Ps) scoring was grouped as shown in Table 5. The TRISS (Ps) ranged from group 0-0.25 to 0.96-1.84 with a mean ± SD score of 0.69 ± 2.288 (Table 2). In the non-survivor (NS) group, 15 patients had TRISS (Ps) 0.26-0.50, followed by 0.51-0.75 (n=18), 0.76-0.90 (n=12) and 0.90-0.95 (n=11). While 16 survivors had TRISS (Ps) between 0.96 and 1. A statistically significant association was found between TRISS and patient outcome (<0.001).

**Table 5:** TRISS (Ps) vs. patient outcome NS: non-survivors; S: survivors

TRISS (Ps)	Patient outcome		Pearson's chi-square test	p-value
	NS	S		
0-0.25	19	0	67.244	<0.001
0.26-0.50	15	0		
0.51-0.75	18	1		
0.76-0.90	12	1		
0.91-0.95	11	1		
0.96-1.00	2	16		
Total	77	19		

Trauma scores vs. patient outcome

Table 6 shows the various scoring systems and outcomes. ISS was significantly higher in the non-surviving (NS) patients, while RTS and TRISS were significantly higher in the surviving (S) group. This shows that the predictive value of survival by TRISS scoring is statistically significant (<0.001).

**Table 6:** Scoring system vs. patient outcome NS: non-survivors; S: survivors

Scores	Patient outcome		Mann-Whitney p-value
	NS median (IQR)	S median (IQR)	
ISS	25 (6)	19 (9)	<0.001
RTS	4.09 (2.5)	6.93 (0.94)	<0.001
RTS Ps	62.8 (54.6)	96.6 (5.5)	<0.001
TRISS	0.18 (2.6)	3.7 (1.3)	<0.001
TRISS Ps	54.6 (56.6)	97.8 (3.2)	<0.001

Trauma scores versus physiological factors

Spearman's correlation coefficient test was used to check the correlation between non-parametric continuous variables. Significant positive correlations were seen between Glasgow Coma Scale (GCS) with RTS, GCS with TRISS, systolic blood pressure (SBP) with RTS, and SBP with TRISS. A significant negative correlation was found between GCS and ISS and between SBP and ISS (Table 7).

**Table 7:** Correlation between trauma scoring systems and vitals GCS: Glasgow Coma Scale; SBP: systolic blood pressure; RR: respiratory rate

Variables	ISS		RTS		TRISS	
	r	p	r	p	r	p
GCS	-0.409	<0.001	0.907	<0.001	0.819	<0.001
SBP	-0.365	<0.001	0.712	<0.001	0.638	<0.001
RR	-0.063	0.543	0.079	0.443	0.101	0.329

Receiver operating characteristic (ROC) curve analysis of trauma scores

**Figure 4:**
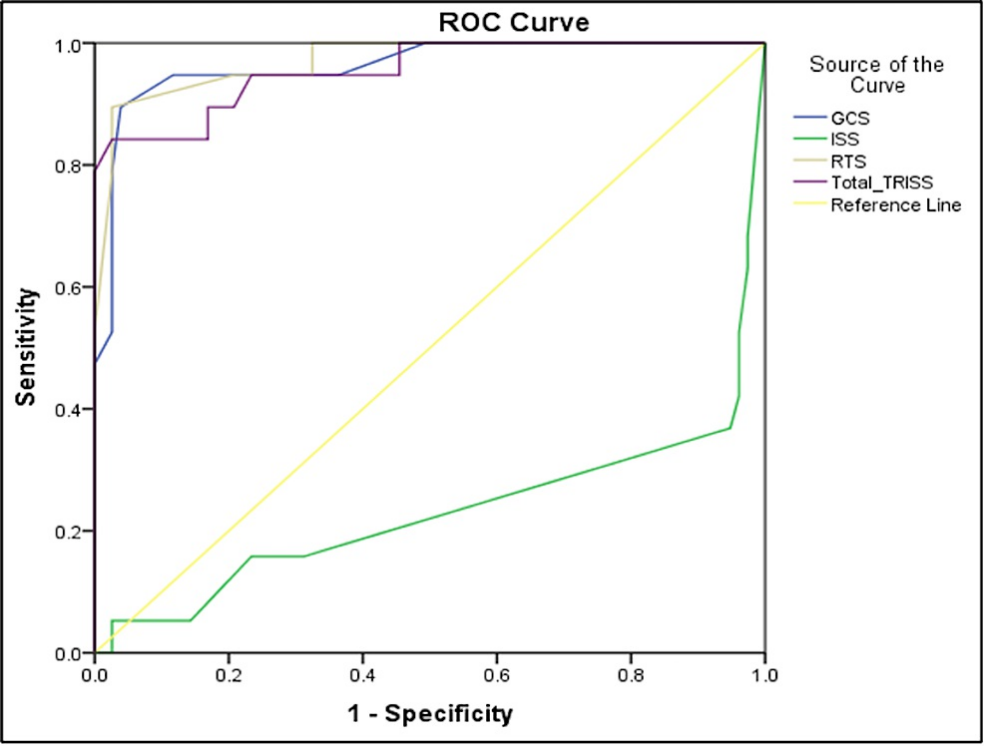
ROC curve for trauma scores

In our study, the ROC curve was plotted to find out the survival predictability using trauma scores after a blunt injury. Figure 4 and Table 8 represent the ROC curve observations for different trauma scores.

**Table 8:** ROC observations

Scoring systems	Cut-off	Sensitivity	Specificity	AUC	p-value
GCS	6.5	94.7	98.8	0.962	<0.001
ISS	22.50	36.8	5.2	0.230	<0.001
RTS	5.932	94.7	79.2	0.971	<0.001
TRISS	1.532	94.7	76.6%	0.955	<0.001
TRISS (Ps)	82.24	94.7	75.3	0.954	<0.001

The area under the ROC curve (AUC) and cut-off scores for ISS, RTS, and TRISS were 0.230 and 22.50, 0.971 and 5.932, 0.955 and 1.532, respectively.

The sensitivity of TRISS (94.7%) was found to be comparable to RTS (94.7%), but the sensitivity was found to be lesser for ISS (36.8%). Specificity was found to be highest for RTS (79.2%) and TRISS (76.6%). The least specific was ISS (5.2%).

Logistic regression analysis

A regression analysis was done to determine how the change in the independent variable caused a significant difference in the dependent variable. The dependent variable is the outcome of trauma patients in our study, and the independent variables are the different trauma scores under study. Logistic regression analysis gave the predictability model for each of the scores. The positive predictive and negative predictive values of each of the scoring systems are given in Table 9. It was observed that GCS, RTS, and TRISS have very high accuracy in predicting the survival of patients (model fit results: chi-square = 64.479, p<0.001).

**Table 9:** Predictive value of trauma scores PPV: positive predictive value; NPV: negative predictive value

Scoring systems	PPV	NPV	Accuracy
GCS	78.9	97.4	93.8
ISS	31.6	97.4	84.4
RTS	78.9	97.4	93.8
TRISS	84.2	97.4	94.8
TRISS (Ps)	84.2	96.1	93.8

A statistically significant model fit result shows that the independent variables, that is, the scores, predict the outcome variable successfully and are of good fit when compared to the no predictor model. The p > 0.05 in the Homer and Lemeshow test shows that the independent variables can predict the outcome, where the unit change in the independent variable will change the outcome by a specific percentage as indicated by the regression coefficient B (Table 10).

**Table 10:** Homer and Lemeshow test

Scores	Regression coefficient (B)	R2	Hosmer and Lemeshow's p-value	Standard error of estimate (SE)	p
GCS	0.751	0.737	0.222	0.174	<0.001
ISS	-0.168	0.193	<0.001	0.059	0.004
RTS	2.727	0.761	0.384	0.740	<0.001
TRISS	2.079	0.722	0.341	0.518	<0.001
TRISS (Ps)	0.185	0.608	<0.001	0.057	<0.001

## Discussion

We used the TRISS score to predict the outcome of trauma in injured patients and the accuracy of various trauma scores.

In the present study, a total of 96 cases of blunt trauma injuries with ISS≥15 were included. We excluded cases of penetrating trauma because most of these patients either died before arrival at the hospital or had a total injury severity score of ISS<15. These findings are not comparable to the results of the previously published studies [17-19]. All 96 cases included had injuries sustained due to blunt trauma. Similarly, in some of the previous studies, most cases (>90%) were of blunt trauma injuries [10,19-21].

In our study, the mean ± SD ISS of all the cases was 27.48 ± 8.79. Non-survivors had a higher mean ± SD and ISS (28.74 ± 8.38) than survivors (22.37 ± 8.8). Pearson’s chi-square test for ISS showed statistically significant differences between survivors and non-survivors (p<0.001).

We found maximum mortality in the ISS group 25-40 (n=63), whereas the ISS group 16-24 had the greatest number of survivors (n=12). Recent studies showed similar findings [8,12,17,22-24]. Garkaz et al. [25] showed a higher mean ISS for non-survivors (56.32 ± 25.02) than our study (28.74 ± 8.38), but the explanations for the findings were not given. However, contrasting findings were noted by Yousefzadeh-Chabok et al. [26]. They found a lower mean ± SD ISS (7.31 ± 6.22) in non-survivors than in survivors (15.95 ± 10.46), although the authors failed to explain the contrasting findings.

One of the main drawbacks of using ISS is that it only includes one score per body region. If there is more than one injury in a body region, it will only include the one with higher severity. Hence, it sometimes underestimates the ability of the score to predict the patient's outcome [27].

We observed that the overall mean ± SD RTS was 4.52 ± 2.08, with survivors having higher mean ± SD RTS scores (7.16 ± 0.9) than non-survivors (3.87 ± 1.75). RTS groups 5 to <6 had a maximum number of non-survivors (n=23), and groups 7 to 7.84 had a maximum number of survivors (n=9). Recent studies have demonstrated comparable results with the results of RTS in our study [10,18,28]. There was a statistically significant difference in the RTS of survivors and non-survivors (Mann-Whitney test p-value <0.001). RTS inherits the limitations of GCS as it is the best verbal response that cannot be assessed in patients who are intubated or mechanically intubated. Patients receiving sedatives or those under the influence of alcohol cannot provide the best response and may overestimate the severity [29]. Factors altering the RR of trauma patients, like the age of the patient, injuries involving the chest, or mechanical ventilation, may also alter the RTS [30].

Demetriades et al. [31] found TRISS to be a good predictor of survival in patients with mild injuries only but not for moderate to major injuries, as it has higher misclassification rates with increasing severity. They also quoted that TRISS calculation is a labour-intensive exercise and serves as a major limitation with minimal benefit. Their findings are in contrast to the findings of this study on TRISS and TRISS (Ps).

In the present study, the overall mean ± SD of TRISS (Ps) was 60.71 ± 32.80, with survivors and non-survivors having mean ± SD of TRISS (Ps) of 94.5 ± 9.5 and 52.38 ± 31.12, respectively. Survivors were more common in groups 0.96-1.00 (n=16), and non-survivors were commonly found in groups 0-0.25 (n=19). 100% mortality was observed in TRISS below 0.26, and with an increase in TRISS, the survivability of patients also increases. Previous studies [10,13,26,28] have shown similar observations. Hence, it can be inferred that TRISS can differentiate between survivors and non-survivors with statistical significance (Mann-Whitney test, p<0.001). However, some of the authors have described the limitations of TRISS in the literature [32], as field triage is difficult with TRISS due to its complex calculation.

Hosseinpour et al. [20] demonstrated a statistically significant correlation (Spearman’s Rho correlation, p<0.001) of trauma scores with GCS and SBP, but no significant correlation was found with RR. These findings were comparable to our study. Similarly, a study by Heydari-Khayat et al. observed a correlation between GCS, SBP, RR, and the first 24 hours of hospitalisation with RTS [33].

The AUROC was > 0.5 for RTS (0.971) and TRISS (0.955). These results were consistent with the results from previous studies [7,8,10,24].

A few previous studies observed very low sensitivity for all three trauma scores [4,34] However, the reason for the observations was not mentioned. Similarly, Can et al. [8] observed low sensitivity for both RTS (30.7) and TRISS (41.6), with ISS showing better sensitivity (62.7) compared to the other two trauma scores. However, the authors did not explain the basis of their results.

In this study, the AUC for ISS was 0.230, with a cut-off score of 22.50 depicting low sensitivity (36.8%) as well as specificity (5.2%), an NPV of 97.4, and a PPV of 31.6. Hence, our findings are in contrast to the findings of previously reported studies (10, 23, 24), which showed higher sensitivity, specificity, and AUROC for ISS.

In this study, seven patients with scores more than the ISS cut-off (22.5) survived, whereas four patients died despite having ISS scores less than the cut-off. TRISS has demonstrated the ability to differentiate between survivors and non-survivors at a ROC cut-off value of 1.532 with sensitivity, specificity, and NPV of 94.7%, 76.6%, and 97.4, respectively. The findings of this study with regards to TRISS and TRISS (Ps) on ROC analysis were compared to the findings of previously reported studies, and their comparisons are shown in Table 11.

**Table 11:** Comparison of ROC observation for TRISS from various studies

Parameter	Our study		Omar et al.^34^	Javali et al.^10^	Saad et al. ^24^	Singh et al.^4^	Can et al. ^8^	Hosseinpour et al. ^20^	Dominengues et al.^19^	Tan et al.^21^
	TRISS	TRISS (Ps)	TRISS (Ps)	TRISS (Ps)	TRISS (Ps)	TRISS (Ps)	TRISS (Ps)	TRISS	TRISS	TRISS
Cut-off	1.532	82.24	NA	91.6	89.75	NA	96	0.36	0.97	0.961
Sensitivity	94.7	94.7	25	97	95.6	21.95	41.6	98.7	80.2	94.14
Specificity	76.6	75.3	76.1	88	72.2	97.5	89.5	77.7	83.7	48.9
AUROC	0.955	0.954	0.957	0.972	NA	NA	0.672	0.988	0.90	0.812

As depicted in Table 11, comparable findings were noted with regard to the ROC analysis of TRISS and TRISS (Ps) in our study compared to the previously reported studies [10, 20-24]. Nevertheless, a few studies reported very low sensitivity for TRISS [4,8]. Contrary to our findings for TRISS, Tan et al. [21] reported less specificity (48.9). Hence, both RTS and TRISS are useful trauma scores for predicting patient outcomes in trauma patients and can be used in the medico-legal assessment of patients in clinical forensic medicine units, as shown in a study by Can et al. [8].

## Conclusions

With its low sensitivity and specificity, ISS was not able to differentiate between survivors and non-survivors. However, RTS and TRISS had high sensitivity and specificity, and AUROC showed significant statistical differences between survivors and non-survivors. The overall performance and accuracy of the TRISS score were found acceptable and can be used to predict the survival of trauma patients.
